# Solvent-Free Synthesis of Flavour Esters through Immobilized Lipase Mediated Transesterification

**DOI:** 10.1155/2013/367410

**Published:** 2013-05-30

**Authors:** Vijay Kumar Garlapati, Rintu Banerjee

**Affiliations:** Microbial Biotechnology and Downstream Processing Laboratory, Agricultural and Food Engineering Department, Indian Institute of Technology, Kharagpur, West Bengal 721302, India

## Abstract

The synthesis of methyl butyrate and octyl acetate through immobilized *Rhizopus oryzae* NRRL 3562 lipase mediated transesterification was studied under solvent-free conditions. The effect of different transesterification variables, namely, molarity of alcohol, reaction time, temperature, agitation, addition of water, and enzyme amount on molar conversion (%) was investigated. A maximum molar conversion of 70.42% and 92.35% was obtained in a reaction time of 14 and 12 h with the transesterification variables of 0.6 M methanol in vinyl butyrate and 2 M octanol in vinyl acetate using 80 U and 60 U immobilized lipase with the agitation speed of 200 rpm and 0.2% water addition at 32°C and 36°C for methyl butyrate and octyl acetate, respectively. The immobilized enzyme has retained good relative activity (more than 95%) up to five and six recycles for methyl butyrate and octyl acetate, respectively. Hence, the present investigation makes a great impingement in natural flavour industry by introducing products synthesized under solvent-free conditions to the flavour market.

## 1. Introduction

Short chain esters often have a characteristic pleasant, fruity odour. Consequently, these esters have notable commercial significance in the fragrance, cosmetics, food, and pharmaceutical industries [1]. Flavour esters produced by extraction from plant and animal sources are not viable due to their presence in minor quantities. Chemical production of flavour esters is not eco-friendly and has some toxic effects on the customer's health. Nowadays, many researchers and industries have switched to biocatalytic flavour synthesis due to consumer's inclination towards natural flavours over chemical ones. These reactions use mild operating conditions, have high specificity with reduced side reactions, and produce high purity flavour compounds by avoiding the expensive separation techniques [2]. Among three different major biotechnological methods (through enzymes, plant cell cultures, and plant tissue cultures), processes employing enzymes are the most common techniques [3].

Methyl butyrate (MB) or methyl ester of butyric acid is an ester with a fruity odour of pineapple, apple, and strawberry. Availing small amounts in plant sources, usually pineapple flavour is produced by distillation of vegetable based essential oils on small scale for utilization as perfumes or food flavours. Octyl acetate (OA) or octyl ethanoate is a flavour ester that is formed from octanol and acetic acid with a fruity orange flavour used in food and beverage industries [4]. Lipase catalyzed esterification and transesterification reactions for flavour esters have numerous food applications such as in the synthesis of modified triacylglycerols, emulsifiers, peptides, and oligosaccharides. Lipases, which are considered to be natural by the food legislation agencies, have been widely investigated for ester synthesis, mainly in organic solvents, due to their enhanced solubility in hydrophobic substrates and elimination of side reactions caused by water [5]. Lipase mediated synthesis of flavour esters under solvent-free conditions (in which the reaction medium involves a reactant itself (i.e., an alcohol) as a solvent) has significant importance in different food and pharmaceutical industries due to the avoidance of toxic solvent and elimination of its recovery during the operation [6]. Lipase catalyzed production of flavour esters by transesterification reactions is influenced by a number of transesterification variables such as molarity of alcohol, reaction time, addition of water, temperature, agitation speed, and amount of immobilized enzyme. Several researchers reported the application of immobilized lipases for the flavour ester synthesis. Lipases were employed for transesterification in organic solvent to produce flavour esters such as isoamyl acetate [7, 8], isoamyl butyrate [9], geranyl acetate [10], citronellyl acetate [11], octyl acetate [12], and methyl butyrate [13]. Akoh and Yee [14] studied the lipase catalyzed transesterification of primary terpene alcohols with vinyl esters in organic media as a solvent. Many works were performed by using immobilized lipases and solvent-free conditions for the synthesis of flavour esters to overcome the problems associated with free enzyme separation and solvent toxicity. Immobilized lipase from *C. rugosa* and porcine pancreatic lipase were employed for the synthesis of isoamyl acetate (banana flavour), ethyl valerate (green apple flavour), and butyl acetate (pineapple flavour) in *n*-hexane [15]. Several authors assessed the immobilized lipases for transesterification ability to produce various flavour esters [16, 17]. Solvent-free synthesis of ethyl oleate reported by Foresti et al. [18], results in a 78.6% conversion in 7 h using *Candida antarctica* B lipase adsorbed on polypropylene powder. In another study Ye et al. [19] synthesized saccharide fatty acid esters in solvent-free conditions and reported 88% yield of fructose oleate.

Based on the present demand and inclination of customers towards natural flavours, the present study has intended to synthesize the flavour esters, namely, methyl butyrate and octyl acetate, through immobilized lipase mediated transesterification under solvent-free conditions.

## 2. Materials and Methods

### 2.1. Immobilized Lipase and Chemicals

Lipase from *Rhizopus oryzae* NRRL 3562 was produced and covalently immobilized on activated silica [20]. *p*-Nitrophenyl palmitate (*p*-NPP), methyl butyrate, and octyl acetate standard were purchased from Sigma (USA). All chemicals used were of AR grade and were procured from Merck, Qualigens, and Himedia, India.

### 2.2. Lipase Assay and Protein Determination

Lipase assay was done spectrophotometrically using *p*-NPP as the substrate [21], and total protein was estimated using modified Lowry method using bovine serum albumin (BSA) as standard [22]. One unit (U) of lipase activity was defined as the amount of lipase that liberates one micromole of *p*-nitrophenol per minute under the standard assay conditions.

### 2.3. Transesterification Reaction

#### 2.3.1. Methyl Butyrate Synthesis

Methyl butyrate synthesis was carried out in screw-capped vials containing 3 mL of different molar concentrations (0.2–10 M) of methanol in vinyl butyrate with different ratios (0.1–10%) of additional water. Reaction was initiated by addition of different units (20−120 U) of immobilized *R. oryzae* NRRL 3562 lipase. Samples were placed for different reaction times (2−20 h) in an orbital shaker at different rpm (100−200 rpm) and temperatures (28−40°C), along with the respective controls (without immobilized lipase). From the reaction mixture, samples were withdrawn at specified time intervals and centrifuged at 1747 g for 10 min to remove the immobilized enzyme. The samples were diluted with *n*-hexane (10 times) and analyzed by gas chromatography.

#### 2.3.2. Octyl Acetate Synthesis

Octyl acetate synthesis was carried out in screw-capped vials containing 3 mL of varying molar concentrations (0.2−10 M) of octanol in vinyl acetate with different ratios (0.1−10%) of additional water. Reaction was initiated by addition of different units (20−120 U) of immobilized *R. oryzae* NRRL 3562 lipase. Samples were placed for a reaction time of 2−20 h in an orbital shaker at different rpm (100−200) and temperatures (28−40°C) along with the respective controls (without immobilized lipase). The reaction samples were collected at specified time intervals and centrifuged at 1747 g for 10 min to remove the immobilized enzyme. The centrifuged samples were diluted with *n*-hexane (10 times) and analyzed by gas chromatography.

### 2.4. GC Analysis

#### 2.4.1. Methyl Butyrate

Synthesis of methyl butyrate was analyzed by injecting the diluted aliquots of the reaction mixture in an Agilent 6820 Gas Chromatograph with a flame-ionization detector (USA). The capillary column (length: 30 m, internal diameter: 0.25 mm) with nitrogen as the carrier gas at a constant pressure of 4 kg cm^2^ was used. Column temperature was kept at 60°C for 1 min and then raised to 220°C at the rate of 10°C. Thereafter, it was raised to 240°C at the rate of 10°C and finally maintained at this temperature for 5 min. The temperatures of the injector and detector were set at 200°C and 265°C, respectively. The retention time of methyl butyrate was 21.2 min. The % molar conversion of product was identified and calculated by comparing the peak areas of standard methyl butyrate at the particular retention time.

#### 2.4.2. Octyl Acetate

The diluted reaction mixture was analyzed for synthesis of octyl acetate by Agilent 6820 Gas Chromatograph with a flame-ionization detector (USA). Nitrogen was used as the carrier gas at a constant pressure of 4 kg cm^2^. The capillary column (length: 30 m, internal diameter: 0.25 mm) was kept at 45°C for 2 min, thereafter raised to 260°C, and maintained at this temperature for 1.63 min. The temperatures of the injector and detector were set at 250°C and 280°C, respectively. The retention time of octyl acetate was 6.7 min. The % molar conversion of product was identified and calculated by comparing the peak areas of standard octyl acetate at the particular retention time.

## 3. Results and Discussion

### 3.1. Effect of Alcohol Molarity on Lipase Catalyzed Flavour Esters

**Figure 1:**
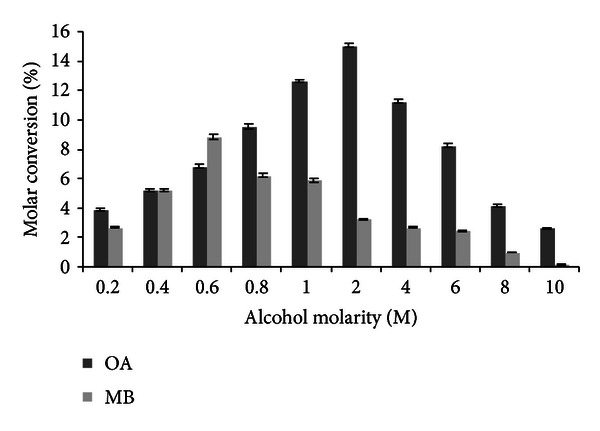
Effect of alcohol molarity on transesterification (experimental conditions: reaction time: 12 h (OA and MB); water addition: 0% (OA and MB); temperature: 32°C (OA and MB); agitation: 100 rpm (OA and MB); enzyme amount: 10 U (OA and MB)). All values are represented as mean ± SD of three replications.

The effect of alcohol molar concentration on molar conversion was investigated in a solvent-free system. As shown in Figure 1, a maximum molar conversion of methyl butyrate and octyl acetate was observed at 0.6 M methanol in vinyl butyrate (1 M theoretical alcohol molarity) and 2 M Octanol in vinyl acetate, respectively. The difference in alcohol molarity towards different products may be attributed to either the steric hindrance or electronic effects of substrates on the immobilized lipase or specificity of immobilized lipase towards the substrates. However, the lower molar conversion at higher molar ratio has been attributed to the inhibitory effect of vinyl acetate and vinyl butyrate on enzyme activity [12, 23]. Increasing the nucleophile (alcohol) concentration is one way of pushing the equilibrium in a forward direction. However, at higher concentrations of alcohol, reaction rate may slow down due to slower diffusion rates of alcohols into the support. Hence, it is necessary to optimize the actual excess nucleophile concentration to be employed in a given reaction [24]. Esterification activity gradually decreased upon increasing the alcohol to acid molar ratio beyond 2 M and 0.6 M in case of octyl acetate and methyl butyrate, respectively, which indicate the inhibitory effect of alcohols on enzyme activity beyond those concentrations. The inhibitory effect of alcohol was also reported by Ghamgui et al. [7], where 64% molar conversion of isoamyl acetate was obtained with 2 M alcohol/acid molar ratio. Further increase in the acid/alcohol molar concentration of *S. simulans* lipase activity was inhibited. In another study by Claon and Akoh [11], molar conversion of citronellyl acetate has been decreased by usage of more than 0.3 M acetic acid.

### 3.2. Effect of Reaction Time on Transesterification Reaction

**Figure 2:**
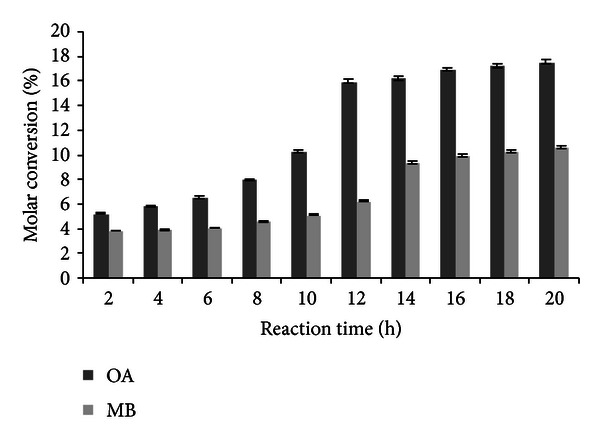
Effect of reaction time on transesterification (experimental conditions: molarity of alcohol: 2 M (OA), 0.6 M (MB); water addition: 0% (OA and MB); temperature: 32°C (OA and MB); agitation: 100 rpm (OA and MB); enzyme amount: 10 U (OA and MB)). All values are represented as mean ± SD of three replications.

Reaction time gives an insight into the performance of an enzyme as the reaction progresses, which will be helpful to determine the shortest time necessary for obtaining good yield and so enhancing cost-effectiveness of the process and will vary with the reaction conditions. In the present study, the effect of reaction time on molar conversion has been shown in Figure 2. The results show that maximum molar conversion has been obtained in reaction times of 14 and 12 h for methyl butyrate and octyl acetate. After the specified time intervals (12 h for octyl acetate and 14 h for methyl butyrate) the molar conversion was relatively constant, which may be due to the attainment of reactions at the equilibrium. Majumder et al. [25] obtained a 100% molar conversion in case of benzyl acetate synthesis in 10 min using Lipozyme RM IM lipase and vinyl acetate as an acyl donor. In another study Bourg-Garros et al. [26] reported 80% bioconversion yield of (Z)-3-hexen-1-yl butyrate in 4 and 6 h using *M. miehi* and *C. antarctica* lipases, respectively, under solvent-free conditions.

### 3.3. Effect of Water Addition on Transesterification Reaction

**Figure 3:**
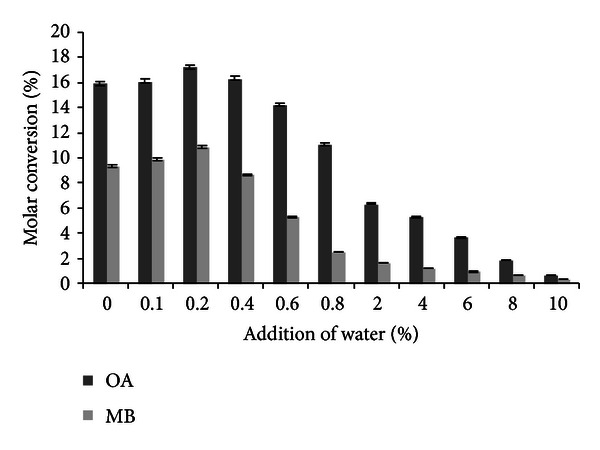
Effect of water addition on molar conversion (experimental conditions: molarity of alcohol: 2 M (OA), 0.6 M (MB); reaction time: 12 h (OA), 14 h (MB); temperature: 32°C (OA and MB); agitation: 100 rpm (OA and MB); enzyme amount: 10 U (OA and MB)). All values are represented as mean ± SD of three replications.

Water has immense importance in lipase mediated reactions both for the maintenance of three dimensional structural integrity and for optimal catalytic activity of the enzyme [27, 28]. The effect of initial water on enzymatic activity was examined by adding water ranging from 0.1% to 10% (v/v) to the reaction mixture. In both cases of flavour esters, addition of 0.2% water in the reaction mixture resulted in a maximum molar conversion (Figure 3). Pepin and Lortie [29] reported the enhanced yields in enantioselective esterification of (R,S)-ibuprofen using *C. antarctica* lipase B in solvent-free conditions using low amount of water in reaction mixture. Denaturation of enzyme activity beyond 1% added water was reported by Bornscheuer et al. [30] in case of chiral resolution of racemic-3-hydroxy ester using *P. cepacia*, *C. viscosum*, and *P. pancreatic* lipases.

### 3.4. Effect of Temperature on Transesterification Reaction

**Figure 4:**
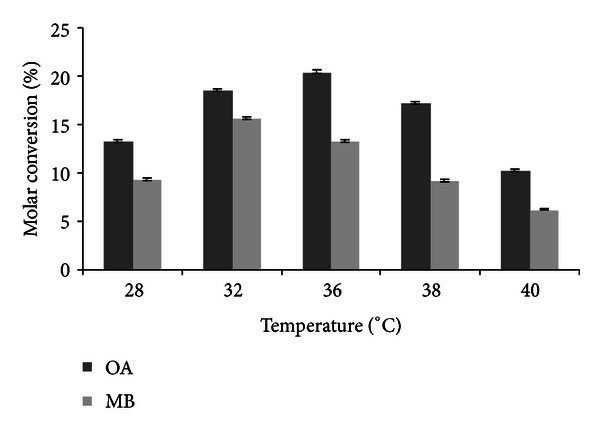
Effect of temperature on transesterification (experimental conditions: molarity of alcohol: 2 M (OA), 0.6 M (MB); reaction time: 12 h (OA), 14 h (MB); water addition: 0.2% (OA and MB); agitation: 100 rpm (OA and MB); enzyme amount: 10 U (OA and MB)). All values are represented as mean ± SD of three replications.

In lipase catalyzed reactions, temperature significantly influences both the initial rate of the reaction and stability of the enzyme. The maximum molar conversion was observed at 32 and 36°C for octyl acetate and methyl butyrate (Figure 4). Welsh et al. [31] reported the inhibition of ethyl butyrate and enhancement of butyl butyrate with the rise in temperature from 30 to 50°C. A yield of 82% octyl acetate was reported by Yadav and Trivedi [12] using Novozyme 435 at 30°C in 90 min.

### 3.5. Effect of Agitation Speed on Transesterification Reaction

**Figure 5:**
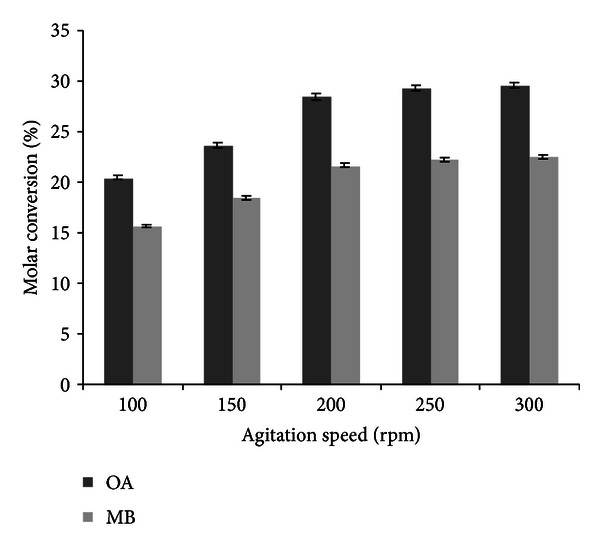
Effect of agitation speed on (experimental conditions: molarity of alcohol: 2 M (OA), 0.6 M (MB); reaction time: 12 h (OA), 14 h (MB); water addition: 0.2% (OA and MB); temperature: 36°C (OA), 32°C (MB); enzyme amount: 10 U (OA and MB)). All values are represented as mean ± SD of three replications.

External mass transfer limitations are generally encountered while working with immobilized enzyme systems. In order to check the external diffusional limitation experimentally, reactions were conducted at different agitation speeds [32]. The effect of speed of agitation was studied in the range of 100−300 rpm (Figure 5). It was observed that the conversion increased with an increase of speed of agitation from 100 to 200 rpm. There was no further change in conversion up to 300 rpm, which indicates that there was no external mass transfer limitation above 200 rpm. Hence, a speed of agitation at 200 rpm was chosen for further studies. At higher agitation rates, the catalyst particles were thrown outside the liquid phase at higher speed, sticking to the wall of the reaction vessel, which would thereby reduce the effective catalyst loading. Also with increasing speed shearing of the enzyme molecule occurs. So if the speed of agitation is increased beyond 200 rpm, molar conversion remains almost unchanged due to shearing of immobilized lipase. Ghamgui et al. [33] reported the maximal molar conversion in case of 1-butyl oleate synthesis by using immobilized *R. oryzae* lipase with an agitation of 200 rpm at 37°C.

### 3.6. Effect of Immobilized Enzyme Amount on Transesterification Reaction

**Figure 6:**
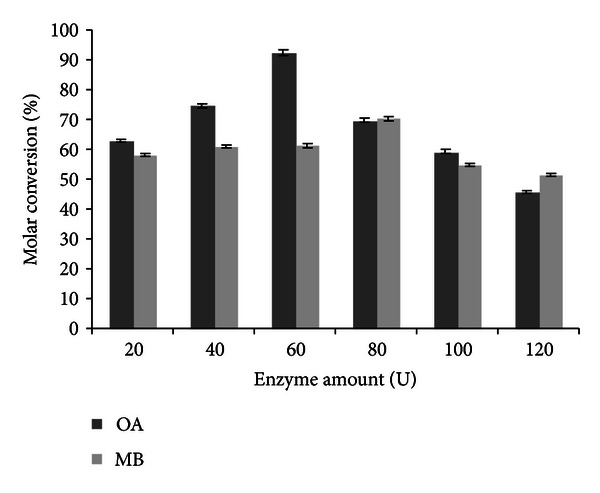
Effect of enzyme amount on transesterification (experimental conditions: molarity of alcohol: 2 M (OA), 0.6 M (MB); reaction time: 12 h (OA), 14 h (MB); water addition: 0.2% (OA and MB); temperature: 36°C (OA), 32°C (MB); agitation: 200 rpm (OA and MB)). All values are represented as mean ± SD of three replications.

The effect of enzyme amount on synthesis of flavour esters was studied by varying the immobilized lipase concentration from 20 U to 120 U. From Figure 6, a molar conversion of 70.42% and 92.35% has been obtained using 80 and 60 U of immobilized lipase for methyl butyrate and octyl acetate, respectively. Upon increasing the enzyme amount further, the molar conversion has been decreased which may be due to difficulty in maintaining uniform suspension of the biocatalysts at higher enzyme concentration due to the agglomeration of immobilized lipase. The excess enzyme did not contribute to the increase in the percentage conversion. The uncontribution behaviour of excess immobilized enzyme amount towards higher molar conversion and its effect on decreased product yield have also been reported in case of amyl isobutyrate [34] and citronellyl acetate [11]. Among all variables, effect of immobilized enzyme amount contributes to attaining higher molar conversions.

### 3.7. Reusability of Immobilized Lipase

**Figure 7:**
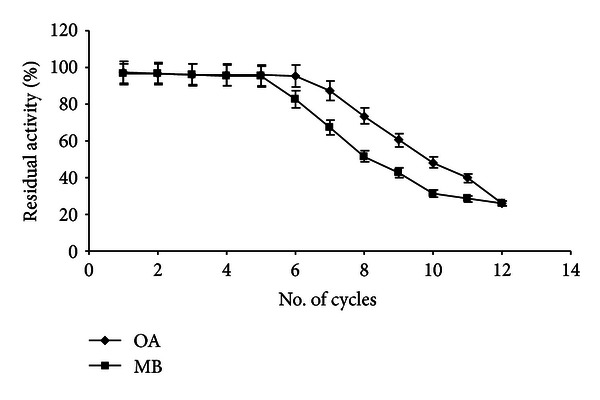
Reusability of immobilized lipase in flavour ester synthesis (experimental conditions: molarity of alcohol: 2 M (OA), 0.6 M (MB); reaction time: 12 h (OA), 14 h (MB); water addition: 0.2% (OA and MB); temperature: 36°C (OA), 32°C (MB); agitation: 200 rpm (OA and MB); enzyme amount: 60 U (OA), 80 (MB)). All values are represented as mean ± SD of three replications.

Reusability studies of the immobilized lipase were carried out by using the recovered enzyme for subsequent cycles. After each cycle, the enzyme was filtered out, washed with t-butanol for regeneration of activity, and allowed to drain out the solvent before reuse. The immobilized enzyme has retained good relative activity (more than 95%) up to five and six recycles for methyl butyrate and octyl acetate, respectively (Figure 7). Chiou and Wu [35] reported the enhanced reusability of *C. rugosa* immobilized lipase on chitosan beads in hydrolytic reaction by retaining its initial activity up to 10 batch hydrolytic cycles. The utilization of t-butanol for enzyme activity regeneration in transesterification reaction was reported by Chen and Hwang [36]. The regenerated enzyme has shown enhanced activity and reusability in transesterification reaction.

## 4. Conclusions

The effect of different variables on immobilized lipase catalyzed flavour esters synthesis has been studied under solvent-free conditions and results in a maximum molar conversion of 70.42% and 92.35% within 14 and 12 h for methyl butyrate and octyl acetate correspondingly. The immobilized lipase was reusable for five and six recycles with retaining the relative activity of more than 95% for methyl butyrate and octyl acetate. These biologically synthesized flavours with the characteristic pineapple (methyl butyrate) and orange (octyl acetate) flavourings will contribute as natural flavour ingredients in food and pharmaceutical formulations.

## References

[1] Berger RG, de Bont JAM, Eggink G, da Fonseca MMR, Gehrke M, Gros JB, Swift K (1999). Biotransformations in the flavour industry. *Handbook of Flavour and Fragance Chemistry*.

[2] Gatfield IL (1995). Enzymatic and microbial generation of flavours. *Perfumer and Flavorist*.

[3] Welsh FW, Muray WD, Williams RE (1989). Microbiological and enzymatic production of flavour and fragrance chemicals. *Critical Reviews in Biotechnology*.

[4] Somogyi LP (1996). The flavour and fragrance industry: serving a global market. *Chemistry and Industry*.

[5] Langrand G, Rondot N, Triantaphylides C, Baratti J (1990). Short chain flavour esters synthesis by microbial lipases. *Biotechnology Letters*.

[6] Güvenç A, Kapucu N, Mehmetoğlu U (2002). The production of isoamyl acetate using immobilized lipases in a solvent-free system. *Process Biochemistry*.

[7] Ghamgui H, Karra-Chaâbouni M, Bezzine S, Miled N, Gargouri Y (2006). Production of isoamyl acetate with immobilized Staphylococcus simulans lipase in a solvent-free system. *Enzyme and Microbial Technology*.

[8] Torres S, Baigorí MD, Swathy SL, Pandey A, Castro GR (2009). Enzymatic synthesis of banana flavour (isoamyl acetate) by *Bacillus licheniformis* S-86 esterase. *Food Research International*.

[9] Hari Krishna S, Karanth NG (2001). Lipase-catalyzed synthesis of isoamyl butyrate: a kinetic study. *Biochimica et Biophysica Acta*.

[10] Claon PA, Akoh CC (1994). Enzymatic synthesis of geranyl acetate in n-hexane with *Candida antarctica* lipases. *Journal of the American Oil Chemists’ Society*.

[11] Claon PA, Akoh CC (1994). Effect of reaction parameters on SP435 lipase-catalyzed synthesis of citronellyl acetate in organic solvent. *Enzyme and Microbial Technology*.

[12] Yadav GD, Trivedi AH (2003). Kinetic modeling of immobilized-lipase catalyzed transesterification of n-octanol with vinyl acetate in non-aqueous media. *Enzyme and Microbial Technology*.

[13] Kwon DY, Hong YJ, Yoon SH (2000). Enantiomeric synthesis of (S)-2-methylbutanoic acid methyl ester, apple flavor, using lipases in organic solvent. *Journal of Agricultural and Food Chemistry*.

[14] Akoh CC, Yee LN (1998). Lipase-catalyzed transesterification of primary terpene alcohols with vinyl esters in organic media. *Journal of Molecular Catalysis B*.

[15] Ozyilmaz G, Gezer E (2010). Production of aroma esters by immobilized *Candida rugosa* and porcine pancreatic lipase into calcium alginate gel. *Journal of Molecular Catalysis B: Enzymatic*.

[16] Dave R, Madamwar D (2006). Esterification in organic solvents by lipase immobilized in polymer of PVA-alginate-boric acid. *Process Biochemistry*.

[17] Silva JES, Jesus PC (2003). Evaluation of the catalytic activity of lipases immobilized on chrysotile for esterification. *Anais da Academia Brasileira de Ciencias*.

[18] Foresti ML, Alimenti GA, Ferreira ML (2005). Interfacial activation and bioimprinting of *Candida rugosa* lipase immobilized on polypropylene: effect on the enzymatic activity in solvent-free ethyl oleate synthesis. *Enzyme and Microbial Technology*.

[19] Ye R, Pyo SH, Hayes DG (2010). Lipase-catalyzed synthesis of saccharide-fatty acid esters using suspensions of saccharide crystals in solvent-free media. *Journal of the American Oil Chemists' Society*.

[20] Kumari A, Mahapatra P, Kumar GV, Banerjee R (2008). Comparative study of thermostabilty and ester synthesis ability of free and immobilized lipases on cross linked silica gel. *Bioprocess and Biosystems Engineering*.

[21] Garlapati VK, Banerjee R (2010). Evolutionary and swarm intelligence-based approaches for optimization of lipase extraction from fermented broth. *Engineering in Life Sciences*.

[22] Lowry OH, Rosebrough NJ, Farr AL, Randall RJ (1951). Protein measurement with the Folin phenol reagent. *Journal of biological chemistry*.

[23] Manohar B, Divakar S (2005). An artificial neural network analysis of porcine pancreas lipase catalysed esterification of anthranilic acid with methanol. *Process Biochemistry*.

[24] Yahya ARM, Anderson WA, Moo-Young M (1998). Ester synthesis in lipase-catalyzed reactions. *Enzyme and Microbial Technology*.

[25] Majumder AB, Singh B, Dutta D, Sadhukhan S, Gupta MN (2006). Lipase catalyzed synthesis of benzyl acetate in solvent-free medium using vinyl acetate as acyl donor. *Bioorganic and Medicinal Chemistry Letters*.

[26] Bourg-Garros S, Razafindramboa N, Pavia AA (1997). Synthesis of (Z)-3-hexen-1-yl butyrate in hexane and solvent-free medium using mucor miehei and *Candida antarctica* lipases. *Journal of the American Oil Chemists’ Society*.

[27] Zaks A, Klibanov AM (1988). The effect of water on enzyme action in organic media. *Journal of Biological Chemistry*.

[28] Goldberg M, Thomas D, Legoy MD (1990). Water activity as a key parameter of synthesis reactions: the example of lipase in biphasic (liquid/solid) media. *Enzyme and Microbial Technology*.

[29] Pepin P, Lortie R (1991). Influence of water activity on the enantioselective esterification of (*R, S*)-ibuprofen by *Candida antarctica* lipase B in solventless media. *Biotechnology and Bioengineering*.

[30] Bornscheuer U, Herar A, Kreye L (1993). Factors affecting the lipase catalyzed transesterification reactions of 3-hydroxy esters in organic solvents. *Tetrahedron Asymmetry*.

[31] Welsh FW, Williams RE, Dawson KH (1990). Lipase mediated synthesis of low molecular weight flavor esters. *Journal of Food Science*.

[32] Foresti ML, Errazu A, Ferreira ML (2005). Effect of several reaction parameters in the solvent-free ethyl oleate synthesis using *Candida rugosa* lipase immobilised on polypropylene. *Biochemical Engineering Journal*.

[33] Ghamgui H, Karra-Chaâbouni M, Gargouri Y (2004). 1-Butyl oleate synthesis by immobilized lipase from *Rhizopus oryzae*: a comparative study between n-hexane and solvent-free system. *Enzyme and Microbial Technology*.

[34] Bezbradica D, Mijin D, Šiler-Marinković S, Knežević Z (2006). The *Candida rugosa* lipase catalyzed synthesis of amyl isobutyrate in organic solvent and solvent-free system: a kinetic study. *Journal of Molecular Catalysis B: Enzymatic*.

[35] Chiou SH, Wu WT (2004). Immobilization of *Candida rugosa* lipase on chitosan with activation of the hydroxyl groups. *Biomaterials*.

[36] Chen JP, Hwang YN (2003). Polyvinyl formal resin plates impregnated with lipase-entrapped sol-gel polymer for flavor ester synthesis. *Enzyme and Microbial Technology*.

